# Cardamonin Modulates Neuropathic Pain through the Possible Involvement of Serotonergic 5-HT1A Receptor Pathway in CCI-Induced Neuropathic Pain Mice Model

**DOI:** 10.3390/molecules26123677

**Published:** 2021-06-16

**Authors:** Nur Khalisah Kaswan, Noor Aishah Binti Mohammed Izham, Tengku Azam Shah Tengku Mohamad, Mohd Roslan Sulaiman, Enoch Kumar Perimal

**Affiliations:** 1 Department of Biomedical Science, Faculty of Medicine and Health Sciences, Universiti Putra Malaysia (UPM), Serdang 43400, Selangor, Malaysia; nrkhalisah95@gmail.com (N.K.K.); nooraishah.mohammedizham271@gmail.com (N.A.B.M.I.); azamshah@upm.edu.my (T.A.S.T.M.); mrs@upm.edu.my (M.R.S.); 2 Centre of Excellence for Nanoscale BioPhotonics, Australian Research Council, University of Adelaide, Adelaide, SA 5005, Australia

**Keywords:** cardamonin, neuropathic pain, serotonin, 5-HT1A, PCPA, chronic constriction injury, CCI, spinal cord and brainstem

## Abstract

Cardamonin, a naturally occurring chalcone isolated from *Alpinia species* has shown to possess strong anti-inflammatory and anti-nociceptive activities. Previous studies have demonstrated that cardamonin exerts antihyperalgesic and antiallodynic properties in chronic constriction injury (CCI)-induced neuropathic pain animal model. However, the mechanisms underlying cardamonin’s effect have yet to be fully understood. The present study aims to investigate the involvement of the serotonergic system in cardamonin induced antihyperalgesic and antiallodynic effects in CCI-induced neuropathic pain mice model. The neuropathic pain symptoms in the CCI mice model were assessed using Hargreaves Plantar test and von-Frey filament test on day 14 post-surgery. Central depletion of serotonin along the descending serotonergic pathway was done using ρ-chlorophenylalanine (PCPA, 100 mg/kg, i.p.), an inhibitor of serotonin synthesis for four consecutive days before cardamonin treatment, and was found to reverse the antihyperalgesic and antiallodynic effect produced by cardamonin. Pretreatment of the mice with several 5-HT receptor subtypes antagonists: methiothepin (5-HT1/6/7_7_ receptor antagonist, 0.1 mg/kg), WAY 100635 (5-HT1A receptor antagonist, 1 mg/kg), isamoltane (5-HT1B receptor antagonist, 2.5 mg/kg), ketanserin (5-HT2A receptor antagonist, 0.3 mg/kg), and ondansetron (5-HT3 receptor antagonist, 0.5 mg/kg) were shown to abolish the effect of cardamonin induced antihyperalgesic and antiallodynic effects. Further evaluation of the 5-HT1A receptor subtype protein expressions reveals that cardamonin significantly upregulated its expression in the brainstem and spinal cord. Our results suggest that the serotonergic pathway is essential for cardamonin to exert its antineuropathic effect in CCI mice through the involvement of the 5-HT1A receptor subtype in the central nervous system.

## 1. Introduction

Cardamonin (2′, 4′-dihydroxy-6′-methoxychalcone) is a naturally derived chalcone that can be found widely in the fruit and rhizomes of *Alpinia species* [1,2]. It has been identified for its beneficial properties towards human health primarily because of its multi-targeting properties [3,4]. The significant role of cardamonin as anti-inflammatory [5,6], antinociceptive [7], antioxidant [8], anticancer, and antiproliferative [9,10] agent has gained remarkable interest to further understand its effect as a potential therapy for other non-communicable diseases. More recently, we have reported that cardamonin also exerts both antihyperalgesic and antiallodynic effects in neuropathic pain mice model [11].

Neuropathic pain is a debilitating chronic condition affecting an estimated 6 to 10% of the general population [12]. Persistent injury to the somatosensory system results in the development of neuropathic pain which is clinically characterized by spontaneous ongoing or shooting pain and augment pain responses after a noxious or non-noxious stimulus [13]. The alteration of the somatosensory system causes a disruption of the sensory signal transmission in the brain and spinal cord [14]. This alteration of the central nervous system in neuropathic pain patients induces hypersensitivity in the affected area, known as hyperalgesia (amplification of pain intensity of noxious stimulus) and allodynia (pain in response to non-noxious stimulus) [15].

The pathophysiology underlying neuropathic pain is not completely known and is considered complex and multifactorial [16]. Numerous factors can lead to neuropathic pain condition including metabolic disorders, trauma, surgery, exposure to drugs, alcohol, toxins, and infections [17]. Patients with neuropathic pain usually experience ongoing or intermittent spontaneous pain, for example, burning, pricking, shooting, or throbbing which may be accompanied by evoked pain particularly to light touch, temperature, and forces [18].

A complex process has been implicated in neuropathic pain conditions including peripheral and central sensitization. Following nerve injury, the alteration of numerous activities within the nervous system lead to the maintenance and development of neuropathic pain. The alterations include enhanced excitatory neurotransmitters activity (glutamate and substance P), pro-inflammatory mediator release (cytokine, tumor necrosis factor α (TNF-α)), and changes of voltage gated and ion channel activities within the peripheral and central nervous system [17,19].

Apart from that, another mechanism underlying the pathological condition of neuropathic pain is the impairment of the descending facilitatory and inhibitory pathways within the peripheral and central nervous system. The abnormalities of these descending modulatory systems can enhance or suppress pain perception [20,21]. One of the most important descending modulatory pathways in pain processing and perception is the monoaminergic system [22]. It mainly consists of serotonergic, noradrenergic, and dopaminergic neurons [22]. Of these neurons, serotonergic system has been studied tremendously due to the bidirectional effects in modulating neuropathic pain conditions [23,24]. The descending control of serotonergic neuron can be facilitatory or inhibitory and the impairment of descending serotonergic neurons is likely to contribute to the promotion and maintenance of chronic pain [25].

Serotonergic neurons mainly synthesizing serotonin (5-HT) as its neurotransmitter [26]. 5-HT can be found widely in the peripheral and central nervous system. The binding of these neurotransmitters to 5-HT receptors is able to influence the spinal processing of nociceptive information exerting facilitatory or inhibitory effects [27]. The descending facilitatory nociceptive system consists of the anterior cingulate cortex (ACC), the rostral ventromedial medulla (RVM), and the dorsal reticular nucleus of the medulla (DRN) [28,29,30]. On the other hand, the descending inhibitory serotonergic system originates from the periaqueductal gray [31], RVM, and the caudal ventrolateral medulla (VLM) [32,33]. The descending inhibition originates from the RVM, suppresses the nociception transmission through its stimulation of the serotonergic neuron, which, in turn, enhances the release of neurotransmitters in the primary afferent neuron along the inhibitory interneuron and in spinal projection neurons [34].

Additionally, different 5-HT receptor subtypes are also implicated in the descending modulation of the serotonergic system. Seven 5-HT families consisting of 15 different subtypes have been identified so far [35,36]. All 5-HT receptors families are G protein-coupled receptors except for 5-HT3 which is an ion gated channel. It has been shown that 5-HT interferes with nociceptive transmission through the activation of multiple neuronal 5-HT receptors at the peripheral site [37,38]. Activation of 5-HT1-3 and 5-HT7 are able to produce antinociceptive effects in pain models [39,40,41]. Conversely, the contradictory report on 5-HT2/3/7 suggest otherwise [42,43,44]. The discrepancies of data reported is probably due to the type of pain involved, route of the antagonist and agonist administration as well as the receptors distribution [41].

The location and action of 5-HT receptors in the descending serotonergic modulatory pathway is important as it is able to generate algesic and analgesic effects in painful conditions. The mechanisms underlying cardamonin’s antihyperalgesic and antiallodynic effects should be further investigated to identify its effectiveness as an analgesic. Considering the importance of the serotonergic pathway in neuropathic pain, the current study was carried out with the main objective to determine the involvement of the serotonergic receptors in the antihyperalgesic and antiallodynic effects of cardamonin and to observe the involvement of the 5-HT1A receptor expression specifically in the cerebral cortex, brainstem, and spinal cord following the treatment.

## 2. Results

### 2.1. Involvement of the 5-HT System in the Antihyperalgesic and Antiallodynic Effects of Cardamonin

**Figure 1:**
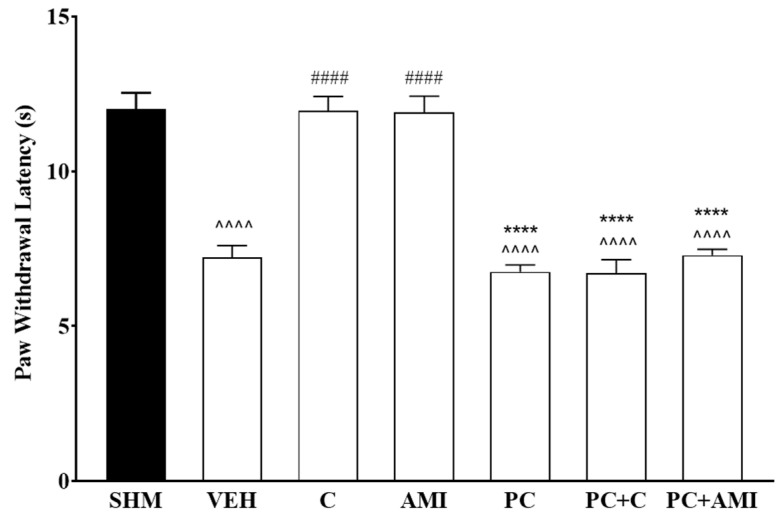
Effect of ρ-chlorophenylalanine (PCPA, a serotonin synthesis inhibitor) pre-treatment on cardamonin against thermal hyperalgesia in CCI-induced neuropathic pain mice, evaluated using Hargreaves’ Plantar test. Data were presented as mean ± SEM (*n* = 6). The caret, hash, and asterisks denote the significance levels (one-way ANOVA, followed by Tukey’s post hoc test, ^^^^^^ *p <* 0.0001 as compared to sham, ^####^ *p <* 0.0001 as compared to vehicle and **** *p <* 0.0001 as compared to cardamonin-treated group. SHM (Sham); VEH (Vehicle, 10 mL/kg i.p.); C (Cardamonin, 10 mg/kg i.p.); AMI (Amitriptyline, 20 mg/kg i.p.); PC (ρ- chlorophenylalanine, 100 mg/kg i.p.).

**Figure 2:**
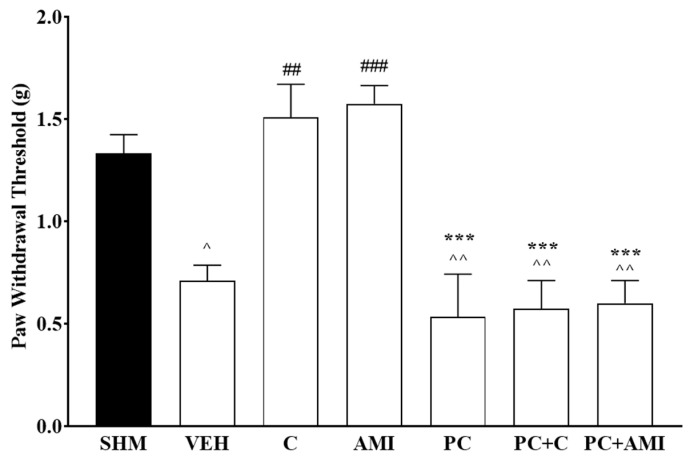
Effect of ρ- chlorophenylalanine (PCPA, a serotonin synthesis inhibitor) pre-treatment on cardamonin against mechanical allodynia of CCI-induced neuropathic pain mice were evaluated using von-Frey filament test. Data were presented as mean ± SEM (*n* = 6). The caret, hash, and asterisks denote the significance levels (one-way ANOVA, followed by Tukey’s post hoc test. ^^^ *p* < 0.05 as compared to the sham, ^^^^ *p <* 0.01 as compared to sham, ^##^ *p <* 0.01, ^###^ *p <* 0.001 as compared to vehicle and *** *p <* 0.001 as compared to cardamonin-treated group. SHM (Sham); VEH (Vehicle, 10 mL/kg i.p.); C (Cardamonin, 10 mg/kg i.p.); AMI (Amitriptyline, 20 mg/kg i.p.); PC (ρ- chlorophenylalanine, 100 mg/kg i.p.).

Participation of the serotonergic system in cardamonin-induced antihyperalgesic and antiallodynic effects were evaluated by pretreating the animals with PCPA (100 mg/kg, i.p.) for four consecutive days. Depletion of central 5-HT after 4 days significantly reversed the antihyperalgesic (F:6,35 = 42.84, *p* < 0.0001) and antiallodynic (F:6,35 = 12.81, *p* < 0.0001) effects in cardamonin-treated groups of CCI mice (Figure 1 and Figure 2). Amitriptyline shows a similar reduction in thermal withdrawal latency and mechanical withdrawal threshold after pre-treatment with PCPA. Serotonin depletion through the administration of PCPA alone did not affect the measurement of hyperalgesia and allodynia in the sham operated and vehicle-treated mice. These results suggested that the antihyperalgesic and antiallodynic properties of cardamonin in CCI-induced neuropathic pain mice were possibly mediated by the descending inhibitory serotonergic system.

### 2.2. Involvement of 5-HT Receptor Subtypes to Induce Antihyperalgesic and Antiallodynic Effects of Cardamonin

Evaluation on the involvement of specific receptor subtypes in cardamonin-induced antineuropathic effect was carried out through the co-administration of cardamonin (10 mg/kg) with serotonin receptor subtypes antagonists. The antihyperalgesic and antiallodynic effects of cardamonin were reversed following the administration of 5-HT1/6/7, 5-HT2A and 5-HT3 receptor antagonists.

**Figure 3:**
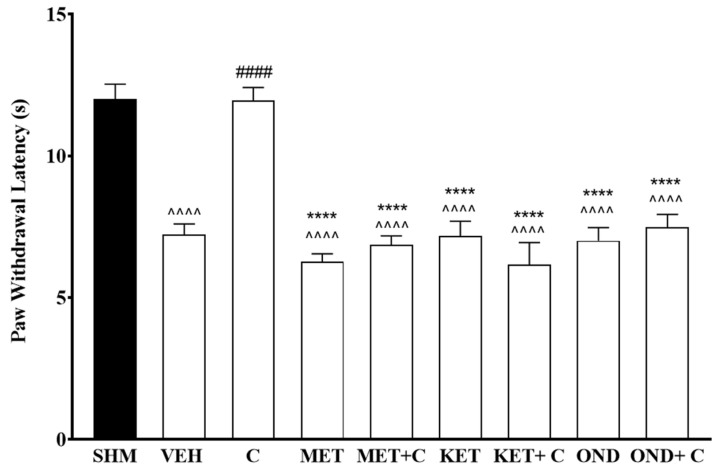
Effect of methiothepin (5-HT1/6/7 receptor antagonist), ketanserin (5-HT2A receptor antagonist), and ondansetron (5-HT3 receptor antagonist) pre-treatment on cardamonin against thermal hyperalgesia of CCI-induced neuropathic pain mice were evaluated using Hargreaves’ Plantar test. Data were presented as mean ± SEM (*n* = 6). The caret, hash, and asterisks denote the significance levels (one-way ANOVA, followed by Tukey’s post hoc test, ^^^^^^ *p <* 0.0001 as compared to sham, ^####^ *p <* 0.0001 as compared to vehicle and **** *p <* 0.0001 as compared to cardamonin-treated group. SHM (Sham); VEH (Vehicle, 10 mL/kg i.p.); C (Cardamonin, 10 mg/kg i.p.); MET (Methiothepin, 0.1 mg/kg, i.p.); KET (Ketanserin, 0.3 mg/kg, i.p.); OND (Ondansetron, 0.5 mg/kg, i.p.).

Administration of different receptor antagonists yields a different significant reduction percentage of paw withdrawal latency (F:8,45 = 22.6, *p* < 0.0001) induced by cardamonin (*p* < 0.05). Cardamonin induced-antihyperalgesic effect was reduced (58%, 61%, and 64%) following the administration of methiothepin, ketanserin, and ondansetron, respectively (Figure 3).

**Figure 4:**
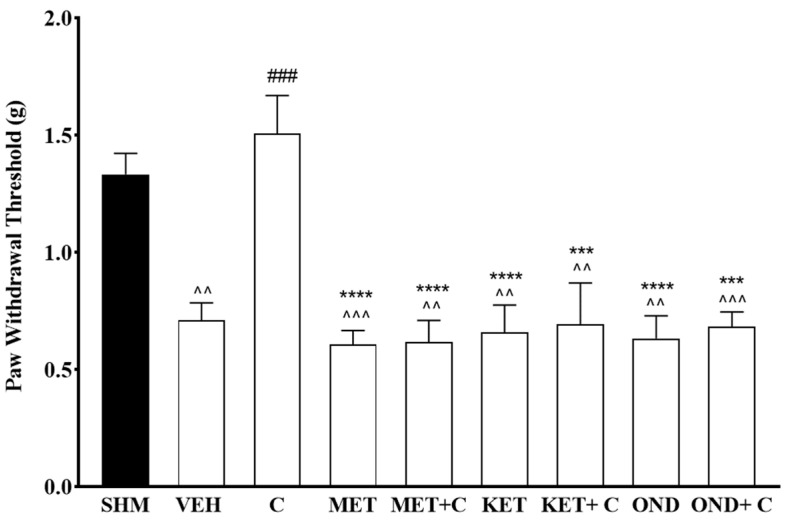
Effect of methiothepin (5-HT1/6/7 receptor antagonist), ketanserin (5-HT2A receptor antagonist), and ondansetron (5-HT3 receptor antagonist) pre-treatment on cardamonin against mechanical allodynia of CCI-induced neuropathic pain mice were evaluated using von-Frey filament test. Data were presented as mean ± SEM (*n* = 6). The caret, hash, and asterisks denote the significance levels (one-way ANOVA, followed by Tukey’s post hoc test, ^^^^ *p <* 0.01, ^^^^^ *p* < 0.001 as compared to sham, ^###^ *p <* 0.001 as compared to vehicle and *** *p <* 0.001, **** *p <* 0.001 as compared to cardamonin-treated group. SHM (Sham); VEH (Vehicle, 10 mL/kg i.p.); C (Cardamonin, 10 mg/kg i.p.); MET (Methiothepin, 0.1 mg/kg, i.p.); KET (Ketanserin, 0.3 mg/kg, i.p.); OND (Ondansetron, 0.5 mg/kg, i.p.).

The reversal of the antiallodynic effect by cardamonin is illustrated in Figure 4. Administration of the receptor agonists prevents re-establishment of paw withdrawal threshold of cardamonin in CCI-induced neuropathic pain mice. The antiallodynic effect were significantly reduced (F:8,45 = 9.528), *p* < 0.0001) when compared to the cardamonin treated group (*p* < 0.05). The results reveal that the administration of methiothepin, ketanserin, and ondansetron were able to reduce cardamonin’s antiallodynic effectiveness up to 38%, 39%, and 45%, respectively. Administration of the receptor antagonists alone did not affect the thermal hyperalgesia and mechanical allodynia in CCI-induced neuropathic pain.

### 2.3. 5-HT1 Receptor Subtype Mediates the Antihyperalgesic and Antiallodynic Effects of Cardamonin

**Figure 5:**
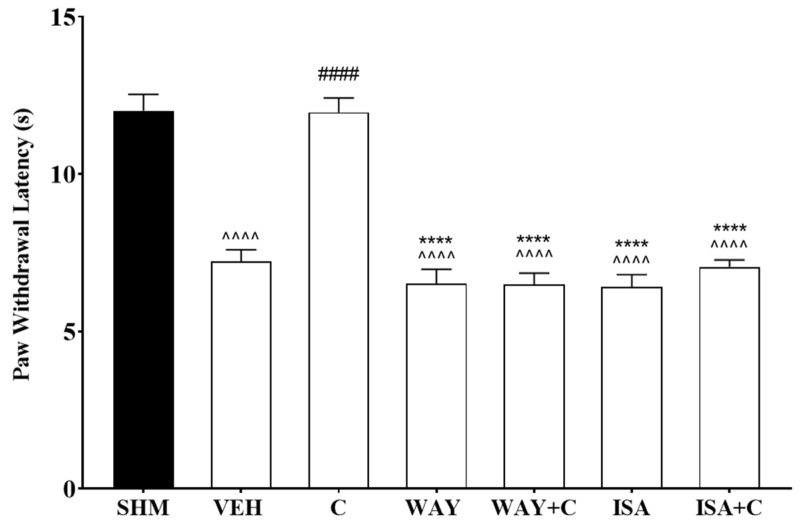
Effect of WAY 100,635 (5-HT1A receptor antagonist) and isamoltane (5-HT1B receptor antagonist) pre-treatment on cardamonin against thermal hyperalgesia of CCI-induced neuropathic pain mice were evaluated using Hargreaves’ Plantar test. Data were presented as mean ± SEM (*n* = 6). The caret, hash, and asterisks denote the significance levels (one-way ANOVA, followed by Tukey’s post hoc test, ^^^^^^ *p <* 0.0001 as compared to sham, ^####^ *p <* 0.0001 as compared to vehicle and **** *p <* 0.0001 as compared to cardamonin-treated group. SHM (Sham); VEH (Vehicle, 10 mL/kg i.p.); C (Cardamonin, 10 mg/kg i.p.); WAY (WAY 100635, 1.0 mg/kg, i.p.); ISA (Isamoltane, 2.5 mg/kg, i.p.).

**Figure 6:**
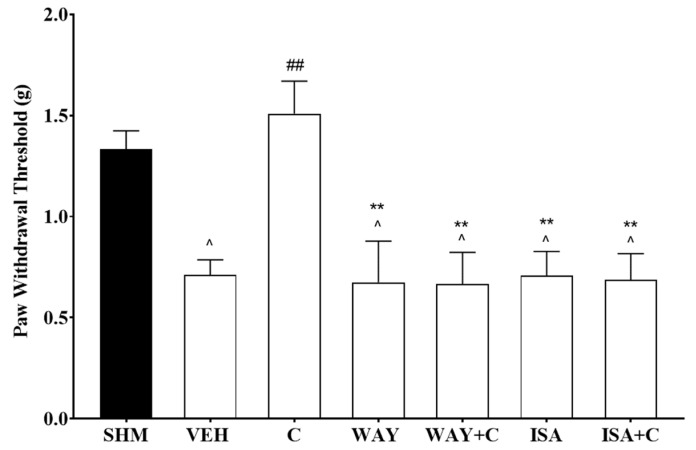
Effect of WAY 100,635 (5-HT1A receptor antagonist) and isamoltane (5-HT1B receptor antagonist) pre-treatment on cardamonin against mechanical allodynia of CCI-induced neuropathic pain mice were evaluated using von-Frey filament test. Data were presented as mean ± SEM (*n* = 6). The caret, hash, and asterisks denote significance level (one-way ANOVA, followed by Tukey’s post hoc test. ^^^ *p <* 0.05 as compared to sham, ^##^ *p <* 0.01 as compared to vehicle and ** *p <* 0.01 as compared to cardamonin-treated group. SHM (Sham); VEH (Vehicle, 10 mL/kg i.p.); C (Cardamonin, 10 mg/kg i.p.); WAY (WAY 100635, 1.0 mg/kg, i.p.); ISA (Isamoltane, 2.5 mg/kg, i.p.).

The role of specific 5-HT1 receptor subtypes on antineuropathic pain effects of cardamonin were further evaluated using WAY 100,635 (5-HT1A receptor antagonist) and isamoltane (5-HT1B receptor antagonist). Co-administration of cardamonin with both antagonists blocked the antihyperalgesic and antiallodynic effects shown in the CCI treated mice. WAY 100,635 and isamoltane administration were shown to significantly increase thermal hyperalgesia (F:6,35 = 40.14, *p* < 0.0001) in CCI-induce mice by 42% and 45%, respectively (Figure 5). Mechanical allodynia in the neuropathic pain mice was also significantly enhanced (F:6,35 = 6.686, *p* < 0.0001) by 72% and 64%, respectively, following the antagonist treatment (Figure 6).

### 2.4. 5-HT1A Receptor Expression in Cardamonin-Treated Neuropathic Pain Mice Model

**Figure 7:**
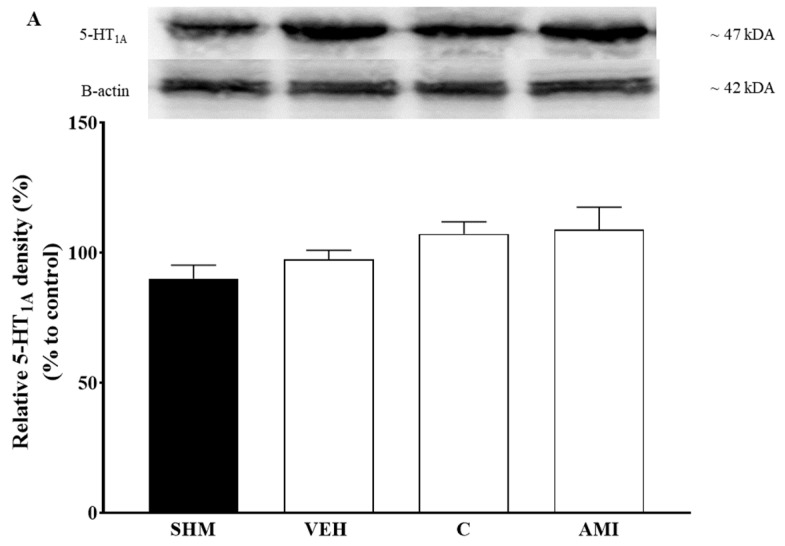

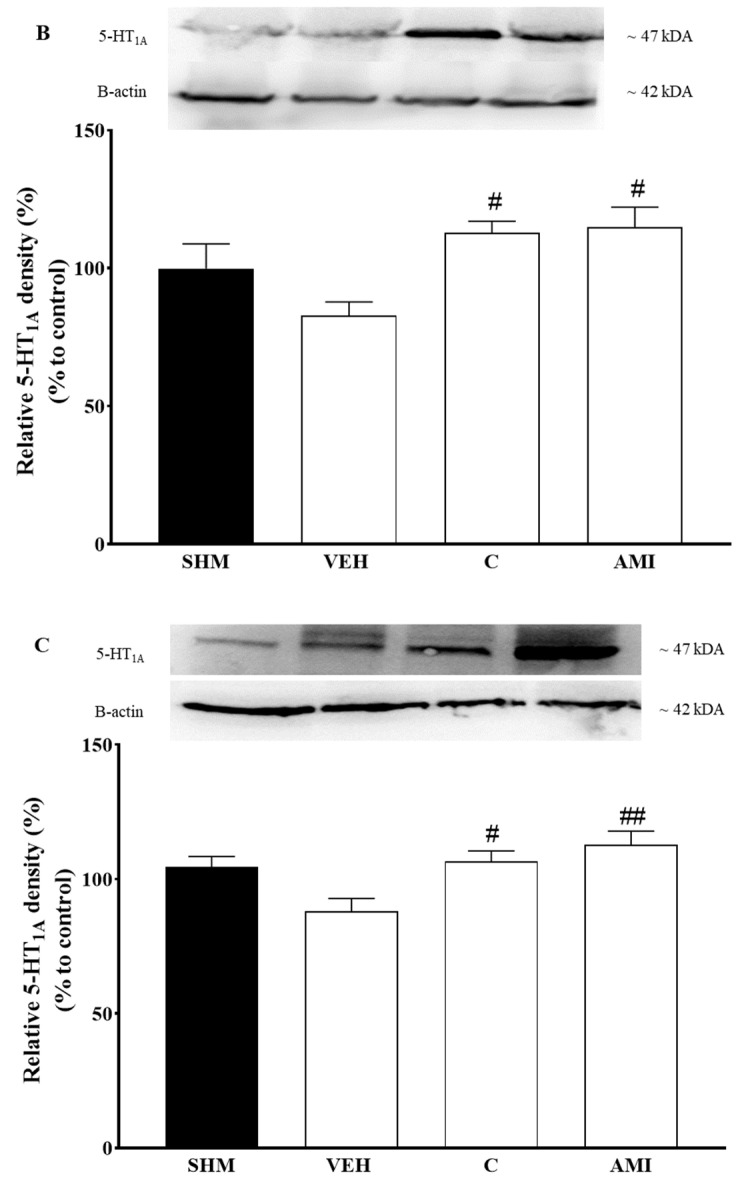
The changes of 5-HT1A protein expression from the: (**A**) Cerebral cortex, (**B**) brainstem, and (**C**) spinal cord were evaluated using western blot. Data presented as mean ± SEM (*n* = 4–5), which were normalized to β-actin. ^#^ *p <* 0.05, ^##^ *p <* 0.01 as compared to vehicle. SHM (Sham); VEH (Vehicle, 10 mL/kg i.p.); C (Cardamonin, 10 mg/kg i.p.); AMI (Amitriptyline, 20 mg/kg i.p.).

Changes in the 5-HT_1A_ protein expression in the cerebral cortex, brainstem, and spinal cord following cardamonin treatment were evaluated using Western blot analysis. CCI-induced animals were shown to downregulate 5-HT1A expression in the brainstem and spinal cord (Figure 7B,C). On the other hand, CCI-induced animals were shown to increase the 5-HT1A receptor expression in the cerebral cortex as shown in Figure 7A. Cardamonin treatment was shown to significantly upregulate 5-HT1A expressions in the spinal cord (F:3, 12 = 5.904 and brainstem (*p* < 0.05) of the CCI-induced neuropathic pain mice as compared to the vehicle group. In contrast, a slight increase of 5-HT1A expression was observed in the cerebral cortex of neuropathic pain mice model. All of the samples revealed bands corresponding to 5-HT1A at ~47 kDA. 

## 3. Discussion

Apart from the fruit and rhizomes of *Alpinia* species, cardamonin is also isolated from various plants of the Zingiberaceae family [4]. Cardamonin is known to possess diverse pharmacological properties against numerous pathological conditions including inflammation [45] and cancer [46]. The multitargeting properties of cardamonin is the key to its therapeutic effects against chronic diseases. Previous studies have demonstrated that cardamonin elicits antihyperalgesic and antiallodynic effects in CCI-induced neuropathic pain mice model modulated via the opioidergic and glutamatergic pathways [11,47,48]. With this understanding, we are now further investigating the possible mechanism of action of cardamonin in neuropathic pain by evaluating the involvement of the serotonergic pathways in cardamonin’s ability to exert antihyperalgesic and antiallodynic properties. 

The present study reveals that cardamonin involves the serotonergic pathway to exhibit antineuropathic in CCI-induced neuropathic pain mice model. More importantly, multiple 5-HT receptor subtypes including 5-HT1A, 5-HT1B, 5-HT2A, 5-HT3, 5-HT6, and 5-HT7 were implicated in mediating cardamonin’s antihyperalgesic and antiallodynic activities. The reversal of mechanical allodynia and thermal hyperalgesia observed following cardamonin treatment were accompanied by the upregulation of 5-HT1A receptor subtype in the spinal cord and brainstem regions. Therefore, we propose that the antihyperalgesic and antiallodynic effects of cardamonin are possibly mediated by the serotonergic pathways and specifically the activation of 5-HT1A receptor subtype as the potential molecular target for cardamonin’s antineuropathic activities.

As previously described, descending monoamines pathways through the serotonergic and noradrenergic neurons were implicated in pain transmission [25,49]. Modulation of the descending monoamines pathway in neuropathic pain were postulated via the innervation of descending serotonergic fibers and noradrenergic fiber from the RVM and locus coeruleus (LC) to the spinal cord, respectively [34,50,51]. Of these, the serotonergic pathway has gained considerable attention compared to the noradrenergic pathway as it is known to have bidirectional effects on pain transmission [23].

Serotonergic neurons release serotonin (5-HT) as its neuromodulator. 5-HT were derived from essential amino acid L-tryptophan, converted into 5-HT via tryptophan hydroxylase (TH) [52]. 5-HT activity in the central and peripheral nervous system can modulate the antinociceptive and nociceptive transmission via descending inhibitory and descending facilitatory pathways, respectively [53,54].

The potential involvement of the serotonergic system in this study was evaluated by depleting the central endogenous 5-HT using a PCPA, a specific serotonin synthesis inhibitor. Continuous endogenous PCPA administration was shown to reduce 65% to 94% of serotonin levels in the brain [55,56,57]. PCPA administration alone does not cause any changes to withdrawal latency and threshold of neuropathic pain animal model; however, the chronic administration of PCPA was able to prevent the analgesic effect produced by other active compounds such as ferulic acid and zerumbone [58,59]. As shown in Figure 1, our study corroborates the previous finding where the administration of PCPA was able to abolish antihyperalgesic and antiallodynic effects produced by cardamonin. Therefore, our data implies that cardamonin is able to alleviate neuropathic pain symptoms by triggering the serotonergic descending inhibitory pathway in CCI-induced neuropathic pain mice.

The bidirectional action of serotonergic neuron is mainly due to the binding of 5-HT to different serotonin receptor subtypes and its localization within the central nervous system [60,61]. Among seven families of serotonin receptors 5-HT1, 5-HT2, 5-HT3, and 5-HT7 receptors were reported to suppress nociception rather than potentiate it [62,63,64].

The involvement of different receptor subtypes in the cardamonin-induced antihyperalgesic and antiallodynic properties were determined using different 5-HT receptor subtype antagonists. Our present findings demonstrated that the administration of both ondansetron (5-HT3) and WAY100635 (5-HT1A) produces the highest reversal of antihyperalgesic and antiallodynic effects in cardamonin-treated mice respectively. The other receptor antagonists such as methiothepin (5-HT1/6/7), isamoltane (5-HT1B), and ketanserin (5-HT2A) also significantly reduced the withdrawal latency and withdrawal threshold effects of cardamonin. Overall, these data suggest that multiple receptor subtypes were involved in mediating antihyperalgesic and antiallodynic effects of cardamonin in CCI-induced neuropathic pain mice.

Our preliminary data using methiothepin demonstrates the involvement of 5-HT1 receptor in cardamonin induced antineuropathic. 5-HT1 is further classified into subfamilies A, B, D, E, and F and the stimulation of 5-HT1 receptor is known to reduce antinociception [20,65,66,67] Endogenous peripheral activation of these subtypes localized on the sensory afferent produces antinociception in animal pain models [67]. In general, 5-HT1 receptors subtypes are coupled to G_i/o_ protein in which the activation of these receptors were able to suppress adenylate cyclase (AC) activity and reduce the cyclic adenosine monophosphate cAMP level, ultimately inactivating protein kinase A [68]. Inhibition of protein kinases were shown to attenuate inflammation and neuropathic pain in different animal models [69].

To further explore 5-HT1 receptor mechanisms of action in cardamonin induced analgesia, the 5-HT1A and 5-HT1B receptor subtypes were evaluated. 5-HT1A was the first serotonergic receptor that has been discovered. It is present abundantly in the human spinal cord and brain at both pre- and post-synaptic regions [70,71]. The 5-HT1A receptor subtype was commonly known to mediate nociception transmission. The activation of 5-HT1A receptors lead to the neuronal hyperpolarization and decreases the firing rate by activating and inhibiting the K^+^ channel and the voltage-gated Ca^2+^ channel, respectively [72,73]. The administration of WAY 100635, a 5-HT1A receptor antagonist, was able to prevent antihyperalgesia and antiallodynia produced by cardamonin [59,74,75]. Our findings agree with previous data where the administration of WAY 100,635 reduces the antihyperalgesic and antiallodynic effects induced by cardamonin supporting the antinociceptive action of 5-HT1A receptors.

Previous studies have demonstrated an inhibitory effect of 5-HT1B receptor in pain modulation [76,77]. Isamoltane (5-HT1B receptor subtypes antagonist) administration before cardamonin significantly abolished antihyperalgesic and antiallodynic action of cardamonin. Activation of 5-HT1B in CNS reduced the 5-HT neurotransmitters release by negatively coupling to adenylyl cyclase and inhibiting the cAMP signaling pathway via Gα_i_ protein. This triggers the opening and closing of K^+^ and Ca^2+^ channels, respectively [78]. Other than regulating 5-HT transmission, 5-HT1B was also known to facilitate other neurotransmitters such as glutamate and acetylcholine equally important in pain transmission [79,80].

5-HT2 receptors are G_q_ coupled protein receptor and are predominantly excitatory at the postsynaptic region The effect of these receptors were modulated through activation of phospholipase C (PLC) [81]. A substantial number of studies show that 5-HT2 exerts anti- and pro-nociceptive influence upon activation [82,83]. The complex influences of 5-HT2 receptors are probably due to the activation of different receptor subtypes 5-HT2A, 5-HT2B, and 5-HT2C in pain models [84]. Of note, these receptors exhibit different cellular location and pharmacological properties [35].

The selective 5-HT2A receptor antagonist ketanserin was used to evaluate the implication of 5-HT2A receptor in cardamonin-induce antihyperalgesic and antiallodynic effects. The location of 5-HT2A receptors in peripheral sensory neuron and spinal motor neuron were assumed to participate in the peripheral and spinal nociceptive cell sensitization. The facilitatory nociceptive influence of 5-HT2A receptors were perhaps due to the sensitization of PAF [85]. In contrast, the activation of this receptor in the spinal motor neuron were able to enhance inhibitory action mediated through the depolarization of motor neurons that evoke the release of GABA interneuron and C-fiber transmission [62]. In this present study, ketanserin administration blocked the antihyperalgesic and antiallodynic effects of cardamonin in the CCI mice model. Considering the unclear mechanism of 5-HT2A receptor in neuropathic pain, we speculate that cardamonin exerts its effect by acting as an inverse agonist to the 5-HT2 receptors. Unlike the other serotonin receptors, 5-HT3 is a ligand gated ion channel that is located widely in the peripheral and central nervous system. Although this receptor has low availability in the central nervous system compared to the other 5-HT receptors, 5-HT3 receptors present abundantly in the GABAergic neurons which are important for pain processing [86]. Similar to the other receptors, activation of 5-HT3 receptors were reported to have a contradictory results in pain transmission [27,42,87]. The contradictory results were probably attributed due to the varying dosage of antagonist and agonist and type of pain models used.

Our present finding highlights that 5-HT3 receptor antagonist administration was able to block the cardamonin-induced antihyperalgesic and antiallodynic effects. These data suggest that the modulation of pain transmission of 5-HT3 receptors was likely due to the localization of this receptor in the cell bodies and terminal endings of the inhibitory interneurons [88]. The rapid depolarization of peripheral and central neurons caused by activation of presynaptic 5-HT3 receptors lead to the increase of cytosolic Ca^2+^ concentration induced by Ca^2+^ influx [89]. This condition may lead to the depolarization of sodium and potassium channels in the postsynaptic neurons and subsequently triggering the release of GABA neurotransmitters in the dorsal horn of spinal cord. The activation of the inhibitory interneurons is able to inhibit the nociceptive transmission [90,91].

Methiothepin is a nonselective 5-HT1, 5-HT6, and 5-HT7 receptor antagonist which act nonspecifically to either of these receptors in the central and peripheral nervous system to elicit nociception or antinociceptive effects. Earlier data stipulated that activation of 5-HT1 and 5-HT7 exhibit antinociception while 5-HT6 facilitates nociception [61,92,93]. Pre-treatment with methiothepin before cardamonin prevented the antineuropathic effects. It appears that the effect of cardamonin are probably mediated via 5-HT1 as discussed earlier and 5-HT7 rather than 5-HT6 receptor subtypes.

Activation of both 5-HT6 and 5-HT7 receptors increases the adenylyl cyclase (AC) levels which in turn elevate cellular cAMP production. This will eventually cause depolarization and nociception [94]. Further understanding of 5-HT6 receptors mechanism in pain is limited due to the lack of available data. In spite of that, few studies reported on the facilitatory influence of this receptor activation in nociception and blocking the receptors lead to analgesic effects in neuropathic pain model [95,96].

On the other hand, the discovery of the inhibitory influence of 5-HT7 receptor activation has gained attention from researchers to further understand the mechanisms of action of this receptor [93]. The localization of 5-HT7 in the superficial layer of the spinal cord dorsal horn drives the regulation of nociceptive transmission [97]. The antinociception action of 5-HT7 receptors were suggested due to its interaction with other neurotransmitters such as GABA and enkaphalin. Thus, the inhibitory tone of 5-HT7 receptors were possibly modulated by the localization of the spinal inhibitory GABAergic and enkaphalinergic interneurons [40,93]. Further studies using a specific receptor antagonist 5-HT6 and 5-HT7 are necessary to provide a better understanding of these receptors effect in cardamonin induced analgesic.

A significant upregulation of 5-HT1A protein expression in the brainstem and spinal cord were observed following cardamonin administration in the CCI- induced neuropathic pain mice. 5-HT1A is a somatodendritic autoreceptor that is important to inhibit cell firing on the dorsal and median raphe nuclei located in brainstem. It is able to modulate 5-HT transmission in the supraspinal level of the spinal cord [53,73] The analgesic effect of serotonergic descending pathway is innervated from the raphe nuclei to the spinal cord [98,99]. The blockade of 5-HT1A autoreceptor in rostroventral medulla (RVM) promote the endogenous 5-HT release to suppress the pain descending pathway subsequently preventing analgesia in neuropathic pain conditions [53,100].

It is postulated that nerve injury caused by CCI were able to downregulate spinal serotonin levels while enhance 5-HT levels at the injured nerve [60,101]. This is corroborating with our finding where the CCI downregulates the 5-HT1A expression in both regions explaining the possible involvement of this receptor subtype in neuropathic pain. The elevation of 5-HT at the peripheral nervous system is known to promote pronociception and activating the spinal 5-HT1A receptors were able to block the inhibitory action of the GABAergic interneuron on the output neurons projecting to periaqueductal gray (PAG). This activation will then promote the brainstem descending inhibitory system and the depression of nociceptive inputs at the spinal cord levels [85,102]. Therefore, our data suggests cardamonin is able to activate serotonergic descending inhibitory pathway originating from the brainstem. The analgesic effect is probably modulated via the upregulation of 5-HT1A receptor subtypes in the RVM. Spinal action of cardamonin on 5-HT1A receptor subtypes, on the others, are likely to mediate antihyperalgesic and antiallodynic effects by inhibiting GABAergic interneuron activities.

Intraperitoneal injection of cardamonin was shown to systematically attenuate hyperalgesia and allodynia in CCI-induced neuropathic pain mice. Nevertheless, the exact mechanism of action is yet to be discovered. Acknowledging the fact that 90% of 5-HT in the body is synthesized by the enteroendocrine cells (EECs) in the gut, it is speculated that the release of 5-HT from this cells might contribute to pain perception via the activation of diverse 5-HT receptor families on the intrinsic and extrinsic afferent nerve fibers [103,104]. It is hypothesized that during inflammation, 5-HT release from the gut increases. The possible mechanisms of action might be mediated via 5-HT3 receptor which subsequently become sensitized or activation of the nociceptors activity in the dorsal root ganglion (DRG) [105,106]. The hypersensitivity of nociceptors following the gut inflammation might be a contributor to neuropathic pain development.

In this present study, it is suggested that cardamonin could possibly exert the antineuropathic effects in mice by regulating the 5-HT availability, directly inhibiting or modifying pain transmission at different levels along the neuraxis. Therefore, taking the previous literatures and current findings into account, we postulate that the analgesic effect of cardamonin is possibly modulated via the serotonergic pathway focusing on the 5-HT regulation and its receptor actions in the CCI-induced neuropathic pain animal model.

## 4. Material and Method

### 4.1. Chemical

Cardamonin or 2,4-dihydroxy-6-methoxyphenyl)-3-phenyl-2-propen-1-one with ≥98% purity and PCPA, ρ-chlorophenylalanine (serotonin synthesis inhibitor) were obtained from Cayman Chemical (Ann Arbor, MI, USA) and HiMedia (Mumbai, India), respectively. Tween 20, 5% DMSO, tribromoethanol, normal saline (0.9% NaCl), iodine solution, and serotonin antagonist subtypes: methiothepin, ketanserin, ondansetron, and amitriptyline were purchased from Sigma-Aldrich (St. Louis, MO, USA).

### 4.2. Materials

The BRILON non-absorbable surgical suture and silk surgical suture were purchased from Vigilenz Medical Devices Sdn. Bhd. and DemeTech (Miami, FL, USA), respectively.

### 4.3. Experimental Animals

Adult ICR male mice, with a body weight between 25 and 35 g, were used in this experiment. Throughout the experiment, they were housed under 12 h light–dark cycles with access to food and water ad libitum. All the protocols and procedures conducted were approved by the Institutional Animal Care and Use Committee (IACUC) (UPM) (Ref: UPM/IACUC/AUP-R024/2019).

### 4.4. Chronic Constriction Injury (CCI)

The CCI model was chosen and conducted according to Bannett and Xie [107] with minor modifications [59]. The procedures were performed under aseptic condition and the animals were anesthetized intraperitoneally with tribomoethanol (250 mg/kg, i.p.). The sciatic nerve of the left hind leg was exposed using blunt dissection. One loose single ligation using chromic silk suture was placed around the sciatic nerve located between biceps femoris and gluteus superficialis muscle until the mice evoke a brief twitch that can be seen in the respective hind limb. Non-absorbable synthetic sutures were used to suture the incision area. Iodine was applied externally at the injury site using a cotton swab to prevent any infection. Sham-operated mice act as a control and no ligation was done.

### 4.5. Drug, Compound Preparation, and Experimental Groupings

Cardamonin was kept in a refrigerator at −20 °C and freshly prepared prior to the experiment by dissolving in dimethylsulfoxide (DMSO), Tween 20, and normal saline (0.99% NaCl) at a ratio of 5:5:90 (*v*/*v*/*v*). Cardamonin was administered intraperitoneally at a concentration of 10 mg/kg based on previous literature [11]. Amitriptyline (20 mg/kg) was used as a positive control in this experiment based on previous literature [11,108]. Sham (SHM) group consists of animals with (incision, but without CCI procedure), Vehicle (VEH) group consists of animals with (incision, CCI and treated with the solvent used to dissolve the compound), C (Cardamonin) group consists of animals with (incision, CCI and treated with cardamonin), and AMI (Amitriptyline) group consists of animals with (incision, CCI and treated with amitriptyline).

### 4.6. Nociceptive Testing

#### 4.6.1. Assessment of Thermal Hyperalgesia

Thermal hyperalgesia was assessed using Hargreaves Plantar apparatus. The mice were placed in a Plexiglas chamber and allowed to acclimatize for 10 min. The infrared from Hargreaves machine was directed to the mid-plantar surface of the hindpaw. The withdrawal time latency was automatically recorded upon the removal of the hind limb. The cut off time of this test was set at 20 s to prevent tissue injury in the mice.

#### 4.6.2. Assessment of Mechanical Allodynia

Mechanical allodynia was assessed using von-Frey filament from Touch Test Sensory Evaluator (North Coast Medical) California. The animals were placed in the Plexiglas chamber on the elevated wire mesh platform. The von-Frey filament was pressed against the mid plantar surface of the hindpaw until the filament buckled and held for a maximum of 3 s. Immediate flinch or sharp withdraw of the paw were considered as a positive response toward the stimulus. The SUDO method was used in this experiment to evaluate the effect of withdrawal threshold as described by Bonin, Bories, and Koninck [109].

### 4.7. Involvement of Serotonergic System

#### 4.7.1. Serotonin Depletion

PCPA, ρ-chlorophenylalanine (100 mg/kg), or vehicle were administered intraperitoneally from day 11 to day 14 post-surgery. Cardamonin (10 mg/kg) or vehicle were administered twenty minutes after the final injection of PCPA. All the thermal hyperalgesia and mechanical allodynia tests were performed after 30 min of cardamonin and vehicle injection. 

#### 4.7.2. Serotonin Antagonists

Three different antagonists were used to identify the involvement of serotonin receptors comprising of Methiothepin (5-HT1/6/7 receptor antagonist, 0.1 mg/kg), WAY 100,635 (5-HT1A receptor antagonist, 1 mg/kg), Isamoltane (5-HT1B receptor antagonists, 2.5 mg/kg), Ketanserin (5-HT2A receptor antagonist, 0.3 mg/kg), and Ondansetron (5-HT3 receptor antagonist, 0.5 mg/kg) were dissolved in normal saline. The antagonists were administered intraperitoneally 30 min prior to cardamonin administration. All of the doses were determined based on previous literatures [58,59,110,111].

### 4.8. Western Blot Analysis

The animals were sacrificed by spinal dislocation on the day 14 for protein expressions analysis. The lumbar segment of the spinal cord (L4-L5) and brainstem region were removed and homogenized in RIPA buffer with protease inhibitor. The lysates were centrifuged at 14,000× *g* for 10 min at 4 °C. The protein quantification has been done using BCA protein assay kits (Thermo Scientific), Waltham, MA, USA. Then, 50 ug of spinal cord protein and 30 ug of brainstem protein were diluted in 2X leammeli buffer and heated at 95 °C for 5 min. The lysates were then separated using 4–20% Tris HCL (10–250 kD) gel from Bio-Rad, Hercules, CA, USA and transferred into polyvinylidene di-fluoride (PVDF) membranes for 2 h. The membranes were blocked with 5% BSA for an hour at room temperature and then incubated overnight with primary antibody (5-HT1A, 1:2000; Elabscience, US; β-actin, 1:5000; Abcam Group, Cambridge, UK). On the next day, the membranes were washed 3 times with TBST 5 min interval and incubated with secondary antibody (anti-goat IgG, 1:5000; Abcam Group, Cambridge, UK) for one hour and visualized with the chemiluminescent reagent. The proteins were quantified using ImageJ and analysis was performed.

### 4.9. Data Analysis

Results were presented as mean ± SEM. Analysis of data was performed using one-way analysis of variance (ANOVA) followed by Tukey’s post hoc test using GraphPad Prism v9.0 software (GraphPad, San Diego, CA, USA). The significance level has been set at *p* < 0.05.

## 5. Conclusions

As a conclusion, our present findings indicate that serotonergic inhibitory pathway is important for cardamonin to exhibit antihyperalgesic and antiallodynic effects in the CCI-induced neuropathic pain mice model use in this study. Multiple serotonin receptor subtypes such as 5-HT receptor 1A, 1B, 2A, 3, 6, and 7 were postulated to mediate the cardamonin effect. In addition, the action of cardamonin on 5-HT1A receptor expression in the brainstem and spinal cord provides a substantial information on the mechanism of action of cardamonin. Supporting the previous findings of cardamonin against neuropathic pain, cardamonin has a promising potential as a therapeutic lead compound for neuropathic pain. Further research into the effect of cardamonin on different descending nociceptive pathways should be conducted to understand the full mechanisms of action of cardamonin in neuropathic pain conditions.

## Data Availability

The data presented in this study are available upon request from the corresponding author.
